# Nano-Optomechanical Resonators Based on Suspended Graphene for Thermal Stress Sensing

**DOI:** 10.3390/s22239068

**Published:** 2022-11-23

**Authors:** Shen Liu, Hang Xiao, Yanping Chen, Peijing Chen, Wenqi Yan, Qiao Lin, Bonan Liu, Xizhen Xu, Yiping Wang, Xiaoyu Weng, Liwei Liu, Junle Qu

**Affiliations:** 1 Key Laboratory of Optoelectronic Devices and Systems of Guangdong Province and Ministry of Education, College of Physics and Optoelectronic Engineering, Shenzhen University, No. 3688, Nanhai Avenue, Shenzhen 518060, China; 2 Shenzhen Key Laboratory of Photonic Devices and Sensing Systems for Internet of Things, Guangdong and Hong Kong Joint Research Centre for Optical Fibre Sensors, Shenzhen University, Shenzhen 518060, China

**Keywords:** optomechanical resonator, thermal stress sensing

## Abstract

Nanomechanical resonators made from suspended graphene combine the properties of ultracompactness and ultrahigh detection sensitivity, making them interesting devices for sensing applications. However, nanomechanical systems can be affected by membrane stress. The present work developed an optomechanical resonator for thermal stress sensing. The proposed resonator consists of a section of hollow core fiber (HCF) and a trampoline graphene–Au membrane. An all-optical system that integrated optical excitation and optical detection was applied. Then, the resonance frequency of the resonator was obtained through this all-optical system. In addition, this system and the resonator were used to detect the membrane’s built-in stress, which depended on the ambient temperature, by monitoring the resonance frequency shift. The results verified that the temperature-induced thermal effect had a significant impact on membrane stress. Temperature sensitivities of 2.2646 kHz/°C and 2.3212 kHz/°C were obtained when the temperature rose and fell, respectively. As such, we believe that this device will be beneficial for the quality monitoring of graphene mechanical resonators.

## 1. Introduction

As exceptional sensing devices, optomechanical resonators have attracted more and more interests in recent years. Nanomechanical devices have the advantages of a small size and light weight, and have been widely used in the measurement of force [1,2], mass [3,4], thermal radiation [5,6] and pressure [7,8] with extremely high sensitivity. There is a particular interest in nanomechanical resonators based on suspended graphene with excellent electrical conductivity, ultrahigh strength [9,10,11] and good thermal conductivity [12,13,14,15]. Thus, graphene is an ideal candidate for fabricating nanomechanical resonators. However, as a two-dimensional membrane material, graphene is inevitably affected by membrane stress, which has a great influence on the performance of the resonator, such as that of its optics and electronics. If the membrane stress is too large, it not only directly causes cracking and peeling of the membrane but also damages the membrane, thereby destroying the resonator. Therefore, it is necessary to study the membrane stress of the resonator [16,17,18]. At present, the common methods for measuring membrane stress mainly include X-ray diffraction, Raman spectroscopy, nanoindentation, etc. However, they also have some insurmountable defects: the X-ray diffraction method is cumbersome in the process of detection and subsequent data processing [19]; with Raman spectroscopy it is difficult to construct strain models [20]; and the nanoindentation method will cause local damage to the sample to be tested [21].

In this work, we propose an optical fiber nano-optomechanical resonator based on multilayer graphene (MLG) for thermal stress measurement. The proposed resonator consists of a section of hollow core fiber and a trampoline graphene–Au membrane. An all-optical system is applied by integrating optical excitation and optical detection, and the resonance frequency of the resonator is easily obtained through this all-optical system. Thus, the built-in stress of the resonator, depending on temperature change, can be detected by observing the resonance frequency shift. By measuring temperature-induced frequency shifts, temperature sensitivities of 2.2646 kHz/°C and 2.3212 kHz/°C within a temperature range of 20~100 °C are obtained when the temperature rises and falls, respectively.

## 2. Principle and Characterization

**Figure 1:**
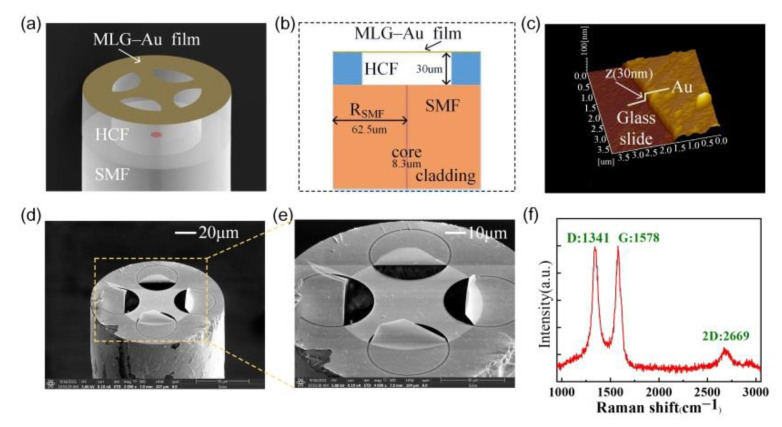
(**a**) Schematic of the membrane-based fiber optic resonator. (**b**) Schematic of side view of the optomechanical resonator. (**c**) Schematic diagram of the glass slide used to measure the thicknesses of the Au film. (**d**) SEM image of the trampoline graphene–Au membrane. (**e**) Enlarged partial view of figure (**d**). (**f**) Raman spectrum from the suspended multilayer graphene (10–15 layers) measured using a 532 nm laser.

In our fiber-optic nanomechanical resonator, as shown in Figure 1a, the temperature of the suspended MLG membrane increases as the ambient temperature increases. Upon absorbing heat, both the membrane and the SiO_2_ will thermally expand. However, due to the graphene and the substrate having different thermal expansion coefficient, i.e., the graphene has a negative thermal expansion coefficient [15] while the substrate has a positive one [22], they will suffer different expansions. In addition, the van der Waals force between graphene and substrate exists [23], allowing the MLG to remain attached to the HCF face [24]. Thus, the built-in stress of the graphene membrane will be changed, resulting in a change in the resonance frequency of the resonator. The thermomechanical stress induced by the temperature increase shifts the resonance frequency of the resonator by an amount [5].

(1) $$\Delta f_{0}=\frac{\alpha Yf_{0}}{2\sigma_{0}(1-\nu )}\Delta T,$$

where α is the thermal expansion coefficient (TEC), *v* is the Poisson ratio, *σ*_0_ is the initial in-plane stress, *Y*, *ƒ*_0_, Δ*T* are the two-dimensional (2D) elastic modulus, the initial frequency and the temperature change, respectively.

The strain due to the TEC mismatch is approximately defined as [17]

(2) $$\varepsilon \left(T\right)=\int_{297}^{T}\left(\alpha_{s}\left(K\right)-\alpha_{mem}\left(K\right)\right)dK,$$

where *α_s_*(*K*) and *α_mem_*(*K*) are the coefficients of thermal expansion of the substrate and graphene, respectively. *ε*(*T*) is measured relative to the strain at room temperature (297 K), the change in stress in the membrane can be described as Δ*σ* = −(*α*Δ*T*)*Y*/(1 − *v*), where *Y* and *v* represent Young’s modulus and the Poisson ratio of graphene, and it is closely related to the membrane stress. Herein, *α*Δ*T* should be replaced by *ε*(*T*) since the stress is caused by the TEC mismatch of the substrate and the membrane.

The relationship between the stress variation caused by the TEC mismatch and the variation is given by an amount

(3) $$\Delta f_{0}=f\left(\sqrt{1+\frac{\Delta \sigma }{\sigma_{0}}}-1\right)\approx f_{0}\left(\frac{\Delta \sigma }{2\sigma_{0}}\right),$$

where Δ*ƒ*_0_ is the frequency change, *ƒ*_0_ is the initial frequency and Δ*σ* and *σ*_0_ are the stress change and the initial in-plane stress, respectively.

In order to secure the integration of the work and provide a clear vision on the measurement principle, the fabrication process of the resonator is presented and introduced in detail below.

First, a section of HCF with an inner diameter of 75 µm was fused to an end-facet of a single-mode optical fiber (SMF, Corning Inc., SMF-28, America) with a commercial fusion splicer (Fujikura, FSM-62S). The other end of the HCF was well-cut with a fixed length of ~30 μm by using a standard fiber cleaver under a microscope system, so as to control the cavity length of the sensor precisely. Then, the MLG membrane was transferred to the end-facet of the HCF using a wet transfer technique reported in [25]. After the transfer, the device was dried in the air for several hours in order to evaporate the water molecule on the surface of the device and make the graphene membrane contact with the surface of the HCF. Then, a layer of Au membrane was deposited on the tip of the sensor via a magnetron sputtering technology to improve the reflectivity of the membrane [26,27]. Third, the graphene–Au membrane on the tip of the sensor was patterned into a trampoline geometry membrane by using a focused ion beam (FIB, FEI Scios2 DualBeam) technology. Finally, the resonator was packaged in a sealed tube with a pressure of 5 × 10^−4^ Pa. The micro-vacuum packaging technology reported in [25] was used to isolate air disturbance.

Figure 1a presents a schematic of a nanomechanical resonator based on a clamped trampoline graphene–Au membrane. The resonator consisted of three parts: a section of SMF, a section of HCF (75 μm internal diameter) and a graphene–Au membrane. The end-facet of the SMF and graphene–Au membrane acted as two reflective mirrors, forming a Fabry–Pérot interferometer (FPI). The length between the two mirrors defined the cavity length, which could be controlled according to requirements by accurately controlling the HCF length. In addition, this cavity length could be calculated from the reflection spectrum. Figure 1b shows a side-view schematic of the resonator, which consisted of an SMF, a section of HCF and a graphene–Au membrane. The graphene–Au membrane suspended on the HCF and the end-facet of the SMF were used as two reflective mirrors, forming a Fabry–Pérot cavity. The cavity between the two reflective surfaces was filled with air. Figure 1c characterizes the thickness of the Au layer on the membrane, which was measured to be about 30 nm thick by using an AFM [28,29]. Figure 1d shows a scanning electron microscopy (SEM) image of the trampoline MLG membrane covered on the fiber end-facet with an inner diameter of 75 µm and the enlarged SEM image of the membrane in Figure 1d is showed in Figure 1e. The Raman spectrum obtained from the graphene after transferring to the fiber end-facet is shown in Figure 1f and its three mode peaks were at 1341, 1578 and 2669 cm^−1^, and the I_D_/I_G_ and I_2D_/I_G_ were 0.99 and 0.25, respectively. The energy difference between the 2D and G peaks corresponded to that expected for a multilayer graphene film [30].

**Figure 2:**
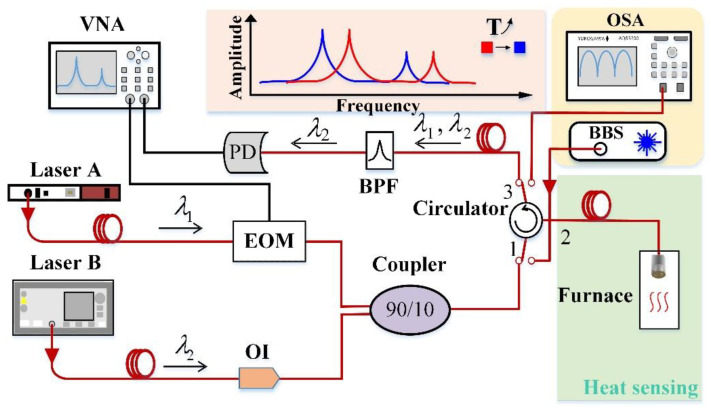
Experimental setup for thermal stress measurement. The all-fiber experimental setup for monitoring the response of a fiber tip resonator to temperature changes and acquiring optical reflection spectra.

Figure 2 shows the diagram of the experimental setup. This all-optical-fiber system integrated optical excitation and optical detection. For the excitation, an intensity-modulated pump laser (Laser A with a wavelength of 1549 nm) with a power of 12 mW and frequency sweeping from 10 kHz to 1.5 MHz was employed to excite the suspended graphene/Au membrane. The pump laser (Knheras ADJUSTIK E15 PM FM, Luster LightTech International Co., Ltd., Denmark) was modulated by an electro-optic modulator (EOM) (Beijing Conquer Optics Science & Technology Co., Ltd., Beijing, China) driven by a vector network analyzer (VNA, R&S^®^ZNL6). Simultaneously, another probe laser (Laser B, Agilent 81940A, America) with a wavelength of 1530.01 nm and a power of 4 mW was employed to detect the mechanical vibrations of the membrane with a maximum sensitivity. These two beams from laser A and B were coupled together through a 90:10 fiber coupler and then coupled into the resonator which was placed in a heating furnace via a three-port circulator. Due to the effect of the frequency-modulated laser A, the graphene membrane on the fiber end-facet thermally expanded or shrank, causing the membrane to resonate. The vibrations generated by the graphene/Au membrane modulated the intensity of the laser B beam reflected from the silica fiber end and the MLG surface. The light reflected from the resonator passed through a tunable band-pass filter (TBF-1550-1.0-FCAPC, Newport, America) to separate the 1549 nm laser and finally, the light intensity of laser B with a wavelength of 1530.01 nm was detected by a photodetector (10 MHz adjustable photoreceiver, model 2053, New Focus, Inc., San Jose, CA, USA) via the circulator. The electric signal from the PD was further analyzed and processed by the VNA. When the frequency of the laser A matched the resonance frequency of the membrane, the amplitude of the membrane vibration was the largest; as such, the reflected light intensity also reached the largest. Therefore, there was a resonance peak on the frequency spectrum. In addition, a typical measurement setup shown in the light-yellow part was used for measuring the reflection spectrum of the resonator. This measurement system consisted of a broadband light source, a circulator and an optical spectrum analyzer (YOKOGAWA AQ6370C) with a resolution of 0.02 nm.

## 3. Result and Discussion

Herein, we propose a way to explore the relationship between the temperature-induced in-plane stress in the membrane and the temperature. As aforementioned, the resonator was microvacuum-packaged, so it was less disturbed by the movement of the external air, and the air damping had also been minimized, resulting in a high signal-to-noise ratio (SNR). The frequency spectrum of the resonator based on the MLG membrane was first measured at room temperature (19.2 °C) and the error on the temperature was ±0.1 °C.

**Figure 3:**
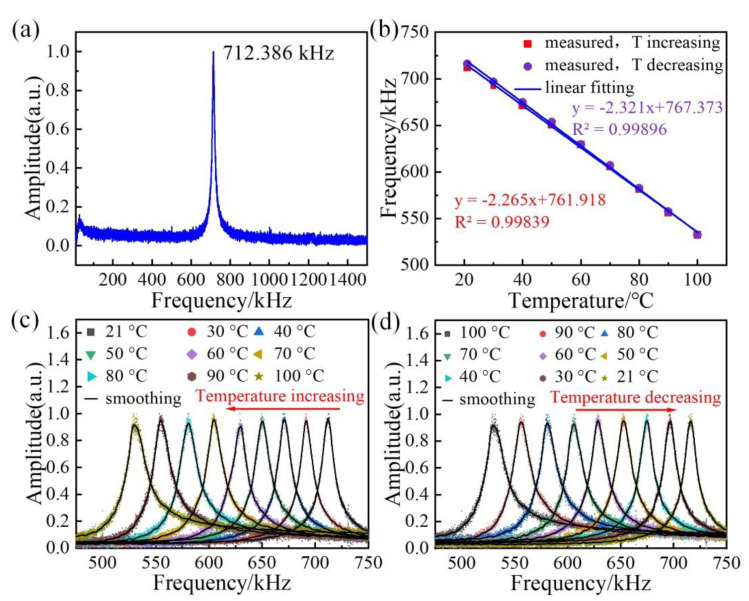
(**a**) Amplitude versus frequency for the fundamental mode of the resonator. (**b**) Resonance frequency ƒ as a function of the ambient temperature T during heating and cooling. (**c**,**d**) Frequency spectral evolution of the resonator, subject to different temperatures.

Figure 3a shows one frequency spectrum of the proposed resonator. The resonance peak was 712.386 kHz with a largest vibration amplitude and the error on the measured frequency was about ±0.001 kHz. This mechanical resonance frequency at the resonance peak was determined by the initial stress of the membrane. The initial stress of this resonator was calculated to be 2.48 MPa by using COMSOL Multiphysics^®^. This resonance frequency was selected for the following test.

To analyze the relationship between the temperature and the built-in stress of the resonator, the fabricated resonator was put into a furnace with a temperature range of 20 °C~100 °C. The resonance frequency shift of the resonator around 712.386 kHz was measured while the temperature was increased from 19.2 °C to 100 °C with an increment step of 10 °C. To ensure a stable furnace temperature, each measurement was recorded 15 min after the temperature setting. Figure 3c,d show the evolution of the resonance frequency spectra as the temperature increases and decreases, respectively. The resonance frequency shifted toward lower frequencies as the temperature increased, due to the built-in stress of the membrane decreases. On the contrary, when the temperature decreased, the built-in stress of the membrane increased, resulting in a higher resonance frequency shift, as shown in Figure 3d.

Figure 3b presents the resonance frequency, ƒ, as a function of the temperature, T, where the red squares indicate the change in resonance frequency as the temperature increases and the purple spheres represent the data as the temperature decreases. As shown in Figure 3b, by linearly fitting the experimental data, it was calculated that the thermal sensitivity was approximately 2.2646 kHz/°C with an R^2^ of 0.99839 during the heating process and the thermal sensitivity was about 2.3212 kHz/°C with an R^2^ of 0.99896 during the cooling process.

**Figure 4:**
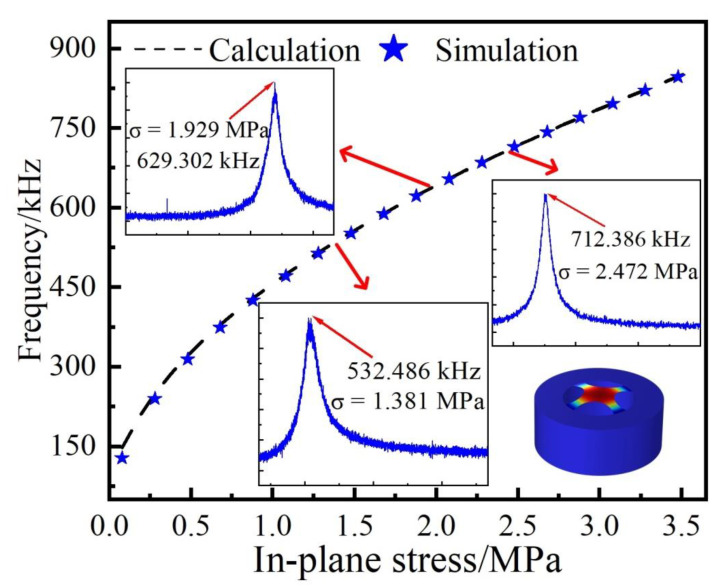
The frequency–stress relationships obtained from COMSOL simulations and calculations, respectively.

In order to investigate the built-in stress of the sensing membrane under the applied temperature, we established a simulation model using COMSOL Multiphysics^®^, using the values of the fabricated resonator, i.e., an inner diameter of 75 µm and a thickness of 3.3 nm [31]. The standard parameters were employed in the simulations, i.e., a graphene density of 233.33 kg/m^3^, Young’s modulus of 1000 GPa, and Poisson’s ratio of 0.186. Figure 4 shows the resonance frequency–stress relationship obtained from COMSOL Multiphysics^®^. The result suggested that the resonance frequency decreased as the built-in stress decreased. Moreover, three measured frequency spectra of the first-order resonance mode under different in-plane stress are shown in the insets in the Figure 4. Thus, we can estimate the membrane built-in stress of the resonator with the measured resonance frequencies. As such, we can obtain the built-in stress change when temperature changes through monitoring the resonance frequency of the resonator. For instance, a built-in stress change of 0.548 MPa was obtained when the two resonance frequencies were measured, i.e., 629.302 kHz and 532.486 kHz. Furthermore, the first-order mechanical resonance mode is shown in the insert.

**Figure 5:**
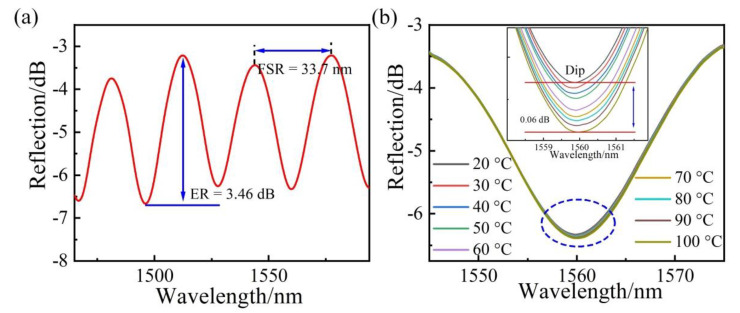
(**a**) Reflection spectra of the MLG-based heat resonator. (**b**) The changes in the reflection spectra at different temperatures and the inset shows the partial enlarged view of the spectrum change.

The built-in stress change of the resonator was also investigated by monitoring the interference spectroscopy. The typical experimental setup is shown in the part circled by the light-yellow part in Figure 2. The light from a broadband source (BBS) was injected into the resonator through a circulator, and then the interference light reflected from the resonator was transmitted to an optical spectrum analyzer (OSA) with a resolution of 0.02 nm. Figure 5a shows the typical reflection spectrum of the resonator, which is related to the cavity length of the resonator. As shown in Figure 5a, the resonance wavelength of one resonance dip was ~1560 nm, the extinction ratio (ER) and the free spectral range (FSR) of the interference spectrum were approximately 3.46 dB and 33.7 nm, respectively. The cavity length and fineness could be calculated to be 36 μm and 1.17, respectively. A working point of 1530.01 nm was employed to detect the mechanical vibrations of the membrane with a maximum sensitivity. The dip wavelength shift of the reflection fringe around ~1560 nm was measured while the temperature was increased from 20 to 100 °C with an increment step of 10 °C. Figure 5b shows the evolution of the resonator reflection spectrum as the temperature increased. The dip wavelength of the reflection spectra at different temperatures are more clearly described in the inset of Figure 5b. The film used for sensing was patterned into a trampoline geometry film and the pressure inside and outside the cavity was consistent. Therefore, the cavity length of the resonator was not affected by temperature. The fringe contrast of the reflection spectra had a decrease of 0.06 dB when temperature increased from 20 °C to 100 °C. Compared with other graphene fiber-optic temperature sensors [32], this obtained cavity length variation was relatively low due to the trampoline design. Therefore, we consider that the change of temperature mainly changes the stress of graphene instead of its cavity length.

## 4. Conclusions

In conclusion, this work demonstrated a nano-optomechanical resonator based on suspended graphene for thermal stress sensing. This resonator consisted of a section of hollow core fiber and a trampoline graphene–Au membrane. Moreover, this resonator was micro-vacuum packaged. This structure could provide high SNR and avoid air thermal expansion. By monitoring the resonance frequency shifts caused by temperature changes, we obtained temperature sensitivities of 2.2646 kHz/°C and 2.3212 kHz/°C for the resonance peak when the temperature rose and fell, respectively. Combining the relationship between resonance frequency and built-in stress, we obtained the built-in stress by simply monitoring the resonance frequency of the resonator. This technology is expected to be used in monitoring the quality of nanoresonators.

## Data Availability

Not applicable.
